# The Role of Adult-Born Neurons in the Constantly Changing Olfactory Bulb Network

**DOI:** 10.1155/2016/1614329

**Published:** 2015-12-29

**Authors:** Sarah Malvaut, Armen Saghatelyan

**Affiliations:** ^1^Cellular Neurobiology Unit, Centre de Recherche de l'Institut Universitaire en Santé Mentale de Québec, Québec City, QC, Canada G1J 2G3; ^2^Department of Psychiatry and Neuroscience, Université Laval, Québec City, QC, Canada G1K 7P4

## Abstract

The adult mammalian brain is remarkably plastic and constantly undergoes structurofunctional modifications in response to environmental stimuli. In many regions plasticity is manifested by modifications in the efficacy of existing synaptic connections or synapse formation and elimination. In a few regions, however, plasticity is brought by the addition of new neurons that integrate into established neuronal networks. This type of neuronal plasticity is particularly prominent in the olfactory bulb (OB) where thousands of neuronal progenitors are produced on a daily basis in the subventricular zone (SVZ) and migrate along the rostral migratory stream (RMS) towards the OB. In the OB, these neuronal precursors differentiate into local interneurons, mature, and functionally integrate into the bulbar network by establishing output synapses with principal neurons. Despite continuous progress, it is still not well understood how normal functioning of the OB is preserved in the constantly remodelling bulbar network and what role adult-born neurons play in odor behaviour. In this review we will discuss different levels of morphofunctional plasticity effected by adult-born neurons and their functional role in the adult OB and also highlight the possibility that different subpopulations of adult-born cells may fulfill distinct functions in the OB neuronal network and odor behaviour.

## 1. Introduction

The olfactory system is essential for the survival of many animal species, providing vital information about food location and influencing social and sexual behaviours. In mammals, odor information is conveyed by olfactory sensory neurons (OSNs) located in the olfactory epithelium. The axon terminals of OSNs establish synaptic contacts in the glomeruli of the OB with mitral cell (MCs). These principal cells then transduce information directly to the olfactory cortex, with no thalamic relay. In the OB, odor information processing is modulated by interneurons: periglomerular cells (PGCs) located in the glomerular layer (GL) and granule cells (GCs) found in the granule cell layer (GCL). GCs are the most abundant population of neurons in the OB and vastly outnumber the bulbar principal neurons by around 100 : 1 [1]. These GABAergic interneurons form a unique type of synapse on the dendrites of principal cells: the dendrodendritic reciprocal synapse in which glutamate, released from the principal cells' dendrites, in turn induces the release of GABA from the spines of interneurons back to the principal cells [2–5]. The two subpopulations of interneurons play an important role in the rhythmic activity of the OB. PGCs coordinate theta activity by regulating baseline and odor-evoked inhibition, whereas GCs are involved in the synchronization of MC activity and generation of gamma rhythm [6–9].

Interestingly, around 10–15% of GCs and 30% of PGCs are continuously renewed during adulthood [10, 11]. This constant renewal affects the distinct subtypes of PGCs and GCs in different ways [12]. In fact, among calretinin, calbindin, and tyrosine hydroxylase (TH) expressing PGCs, the production of calbindin- and TH-positive cells tends to decrease after birth [12, 13], while calretinin expressing-PGCs are mostly produced during adulthood [12]. With regard to GCs, the generation of different subpopulations has not yet been systemically examined and renewal in the adulthood has been shown only for calretinin-expressing, mGluR2-expressing, and small numbers of 5T4-expressing GC subpopulations [12, 14–16]. In general, less is known about the neurochemical heterogeneity of GCs and until now, studies aimed at understanding the role of adult-born neurons in odor information processing and olfactory behaviour have considered these cells as homogenous population of neurons [17–21]. It is conceivable, however, that different subtypes of new neurons may be activated by distinct olfactory tasks and play a specific role in different odor behaviours. There is consequently a need to understand the exact contribution of specific subpopulations of adult-born neurons to animal behaviour.

In this review, after presenting adult OB neurogenesis as a remarkable form of neuronal plasticity, we will discuss recent data concerning the functional role of adult-born neurons and their implications in olfactory behaviour. Finally, we will also discuss the heterogeneity of adult-born neurons and highlight the possibility that different subtypes of new neurons may play distinct roles in the function of the OB network and odor behaviour.

## 2. Adult OB Neurogenesis, an Unusual Form of Structural and Functional Plasticity

New neurons for the OB are produced in the subventricular zone (SVZ), bordering the lateral ventricles, where adult neural stem cells proliferate to give rise to rapidly dividing transient amplifying cells, which in turn divide to produce neuroblasts [22–24]. Neuroblasts migrate tangentially in chains, along the blood vessels through the rostral migratory stream (RMS) towards the OB [25–27]. Once in the OB, they start to migrate radially [28–30], reach maturity, and integrate into preexisting neuronal networks [5, 31–34]. The vast majority of adult-born neurons, around 97%, differentiate into GCs, while the other 3% become PGCs [35].

The constant neuronal turnover occurring in the OB imposes on the olfactory system the never-ending challenge of preserving normal odor information processing despite persistent morphofunctional changes in the large number of interneurons and their synaptic connections with principal cells. The mechanisms enabling the maintenance of this fine equilibrium between normal OB function and the constant rewiring of its neuronal network are not well understood. These mechanisms are driven, however, in an activity-dependent manner and sensory stimulation plays an important role in the maturation, survival, and integration of new neurons in the OB [21, 34, 36–43]. Adult-born neurons may adjust the morphofunctional properties of the bulbar network at several different levels. Firstly, the addition of new neurons may be considered as the topmost level of plasticity along with the continuous formation of new functional synaptic units with principal cells that replace existing connections. Secondly, adult-born neurons may bring to the OB properties that are not present in preexisting interneurons, which may allow them to adapt neuronal network to changing environmental conditions. Finally, new neurons may change their own morphofunctional properties in response to external stimuli to uphold the functioning of the bulbar network.

### 2.1. Plasticity in the OB Network by the Addition of New Neurons

The adult OB is constantly supplied with new cells to be integrated into the preexisting neuronal network.* In vivo* calcium imaging has shown that adult-born PGCs begin to express spontaneous and odor-evoked responses soon after their arrival in the OB network, even if they have the molecular phenotype of immature cells [44]. In addition, other* in vivo* time-lapse imaging studies have shown that new interneurons remain structurally dynamic not only during their maturation and integration phases [44, 45], but also well beyond their synaptic integration [46]. New spines are constantly forming, retracting, and stabilizing on the dendrites of adult-born cells, and even six months after their birth these cells have a higher synaptic density than their preexisting counterparts [47]. Moreover, young adult-born neurons become more selective to particular odors as they mature [48]. These data suggest that adult-born neurons are continuously rewiring the bulbar network and represent a population of cells enabling the OB to adapt to an everchanging odor environment.

In accordance with this, it has recently been demonstrated that the lack of neurogenesis in the adult OB alters the anatomical organization of the bulbar network by modifying the precision and size of intrabulbar projections of principal cells [49]. Suppression of adult neurogenesis has also been shown to affect synchronized activity of MCs* in vitro* [19]. Adult-born neurons provide an important inhibitory input to the bulbar principal cells [19] and* in vivo *experiments have shown that a disruption to the excitation/inhibition balance received by MCs deregulates the ability of animals to discriminate between odors [50]. Thus, the constant supply of new neurons to the OB is a form of morphofunctional plasticity, allowing permanent reorganization of the bulbar network to suit changing environmental conditions.

### 2.2. Adult-Born Neurons, a Population of Cells Remarkably Different from Their Preexisting Counterparts

During maturation, adult-born GCs receive synaptic inputs on the soma and the proximal part of their apical dendrites before forming their output dendrodendritic synapses [5, 51]. This maturational profile of adult-born GCs distinguishes them from their preexisting counterparts that form input and output synapses simultaneously. The sequential acquisition of input synapses before forming the output ones allows these cells to “silently” integrate into the operational bulbar network [51]. However, when adult-born neurons are fully mature they display greater excitability than early-born ones [31, 32]. This increased excitability of adult-born cells enhances their overall inhibitory drive on the principal cells. This is supported by data which shows that ablation of neurogenesis for 28 days results in a decrease of around 45% in the inhibitory input received by MCs [19]. In addition, synapses of adult-born interneurons impinging on the dendrites of principal cells differ from those made by preexisting interneurons in terms of sensitivity to GABA(B) receptors-induced suppression of GABA release [52]. This is due to the different localization of GABA(B) receptors which confers adult-born GCs with synapses different from their preexisting counterparts [52]. These data suggest that adult-born neurons provide stronger inhibitory modulation of the principal cells and have unique features that distinguish them from early-born neurons.

At two to three weeks after birth, adult-born neurons have also been shown to be more responsive to incoming new odors than their older counterparts [53, 54]. This increased responsiveness of adult-born neurons during their development may be due to the critical period during which adult-born cells (at least the PGCs) are less selective to odorant stimuli [48]. Odor selectivity of these cells can be increased by housing animals in an olfactory enriched environment, which suggests that activity-dependent mechanisms play a part in the functional maturation of these cells [48]. During this critical period adult-born cells also display long-term potentiation (LTP) at the glutamatergic input impinging on the proximal dendrites of GCs [55].

Altogether, the results presented above demonstrate that the adult OB receives a population of interneurons distinguishable from the neurons produced during early development and that these cells play a unique role in olfactory information processing.

### 2.3. Plasticity through Changing Morphofunctional Properties of New Neurons

OB neurogenesis is a process sensitive to the level of sensory experience. Several studies have shown that exposing animals to an olfactory enriched environment [39–41] or learning of an associative olfactory task [21, 36] increases survival of adult-born neurons in the OB. Conversely, depriving animals of olfactory input by closing one of their nostrils reduces survival of new neurons [34, 37, 42, 43]. Sensory activity is essential for the development and expression of dopaminergic phenotypes, but not GABAergic, calretinin-, or calbindin-positive ones, suggesting that the acquisition and maintenance of chemo-specific phenotypes by different PGCs can be affected differently by odor-induced activity [56, 57]. It has recently been proposed that mechanisms exist to allow the replacement of preexisting neurons of a particular phenotype, by newborn cells of the same phenotype, thereby maintaining in the OB a constant level of GCs of the same neurochemical subset [15]. In fact, at least for the population of mGluR2-expressing cells, ablation of preexisting GCs of this particular phenotype resulted in a recovery of the density of cells belonging to this subtype [15]. Moreover, adult-born mGluR2-expressing adult-born GCs presented enlarged spines, a characteristic that may aid in compensating for the loss of cells of the same subpopulation [15].

Sensory stimulation is required not only for the survival of new neurons, but also for their maturation, since sensory enrichment enhances synaptogenesis of PGCs [46], whereas sensory deprivation affects the density of spines on the distal dendrites of adult-born GCs [42, 58]. Interestingly, this decreased spine density on the distal dendrites of GCs is accompanied by increased spine density on the apical dendrites [58]. These data suggest a form of structural plasticity among distinct compartments of adult-born GCs that may allow cells to receive stronger input to compensate for a decreased number of output synapses. Further evidence for activity-dependent modifications in the morphofunctional properties of adult-born neurons is observations showing that sensory deprivation triggers an increase in the excitability of new neurons [42]. These sensory-deprivation-induced alterations in the morphology and function of GCs are specific to the adult-born population, since sensory deprivation does not affect the maturation and excitability of preexisting neurons [42]. To understand whether odor stimulation provides a specific pattern of activity or overall membrane depolarization to adult-born neurons which may be required for the survival and maturation of these cells, Lin and colleagues modulated the intrinsic activity of new neurons via overexpression of Kir2.1 potassium and bacterial sodium channels and assessed the survival and integration of new cells in the OB network [59]. These experiments revealed that the intrinsic activity of adult-born neurons plays an important role in their survival but not in their synaptogenesis [59]. Therefore, the plasticity in the morphofunctional properties of adult-born cells themselves may be used to uphold the function of these cells in the OB and allow adjustment of the bulbar network to environmental stimuli. The above data reveal that neurogenesis in the adult OB is tightly linked to sensory experience and represents an important process by which the functioning of the OB network is adjusted to the constantly changing olfactory environment. This is accomplished by different levels of morphofunctional plasticity that are brought by adult-born neurons to the bulbar network.

## 3. Implication of Adult-Born Neurons in Olfactory Behaviour

The fact that a substantial number of adult-born neurons are integrated into the OB neuronal network on a daily basis suggests that these cells may contribute significantly to olfactory behaviour. In this review we will discuss the role of adult-born neurons in spontaneous odor exploration, odor discrimination, and associative memory tasks. The involvement of adult neurogenesis in odor-based social interactions has been discussed elsewhere [60–62] and will not be covered here.

A number of studies have been aimed at determining the potential role of adult-born neurons in various spontaneous odor behaviour tasks. First, odor enrichment [39–41] that increased the survival of adult-born neurons in the OB also resulted in an improvement of short-term odor memory [40, 41]. Irradiation which decreased the number of adult-born neurons produced, however, no change in short-term odor memory [63]. This is likely due to a compensatory increase in the survival of the preexisting population of GCs [63]. Indeed, complete suppression of adult OB neurogenesis using antimitotic drug cytosine *β*-D-arabinofuranoside (AraC), which had no effect on GCs born before AraC infusion, led to impaired short-term olfactory memory [19]. It has also been demonstrated that blocking adult neurogenesis with an antimitotic drug during odor enrichment prevents the enhancement in discrimination abilities of animals [39], supporting the involvement of neurogenesis in the ability to discriminate between similar odors. It appears that adult-born cells are involved specifically in the discrimination of similar odors, since inhibition of neurogenesis with an antimitotic drug had no effect on the ability of mice to discriminate between dissimilar odorants [19]. This was recently confirmed in another study which took advantage of the optogenetic approach to reveal that the specific activation of adult-born neurons facilitates animals' learning to discriminate between two odors, but only when the task is difficult, involving perceptually similar odorants [17]. What are the cellular mechanisms underlying these behavioural modulations? One possibility, as highlighted by electrophysiological recordings at the cellular and network levels following ablation of adult-born cells, is a reduced number of inhibitory synapses made by adult-born GCs on principal cell dendrites which altered inhibitory input and affected synchronized oscillatory activity of principal neurons [19]. Moreover, an optogenetic study has shown that the activation of adult-born neurons improves animal performances in an operant discrimination task of two perceptually similar odorants [17]. It is likely that activation of GCs increases the contrast between MCs since light stimulation triggers an inhibition of MCs with a low firing rate [17]. While these studies highlight some mechanisms of affected odor performances following manipulation in the number or activity of adult-born neurons, there is still a need to better understand the cellular and network mechanisms underlying the role of adult-born interneurons in the processing of olfactory information.

Associative olfactory learning, which evaluates the capacity of an animal to associate an odor with reward or noxious stimuli, is another test widely used to evaluate the implications of adult-born neurons in odor behaviour. Paradigms such as associative olfactory learning, especially those employing perceptually dissimilar odorants increase the survival of adult-born cells in the OB [21, 36, 53, 64]. However, modulation of the levels of neurogenesis had mixed consequences for animals' ability to learn an associative memory task, in some cases having no effect [19, 20], while in others performances were clearly altered [21, 63]. A possible explanation for such differences could stem from the type of odor associative tasks used in these studies [60, 65]. In associative learning procedures, it is possible to differentiate operant conditioning tasks from nonoperant conditioning ones. With operant conditioning tasks, the active behaviour adopted by animals during the learning phase determines whether or not they receive a reward. On the other hand, in nonoperant conditioning tasks, during the learning phase animals make a passive association between the odorant stimuli and the reward. Indeed, Mandairon and colleagues have shown that adult-born neurons play an important role only in operant conditioning tasks, since only these types of task seem to have an impact on the level of neurogenesis [66].

Another question to take into consideration, which may also explain discrepancies in the literature, is whether adult-born neurons are involved in the acquisition, retrieval, and storage of odor-associated memories. Several studies have shown that pharmacological or genetic suppression of adult neurogenesis prior to the task of acquisition does not affect animals' performances [18–21], suggesting that adult-born neurons are not involved in the acquisition of odor-associated memories. Recent observations using a transgenic mouse line based on the “tag-and-ablate” strategy showed, however, that adult-born neurons are involved in the expression of odor-reward memories [18]. In this model, a tamoxifen treatment makes it possible to target or “tag” a large population of adult-born neurons with diphtheria toxin receptor in nestin-positive cells and their progeny. This is followed by a second treatment with diphtheria toxin that ablates the entire tagged cell population. The advantage of this model compared to general adult neurogenesis suppression studies is that adult-born neurons can be specifically ablated during particular phases of memory acquisition, retrieval, or storage. Using this model, the authors demonstrated that in an associative learning task, the ablation of mature newborn neurons just after the training phase resulted in altered olfactory memory. Although this study shows that bulbar adult-born neurons play a key role in the expression of odor-reward memories, this effect appears to be transient. Ablation of adult-born GCs 28 days after training did not produce a memory deficit, suggesting that, with time, recall of odor-reward memory becomes independent from adult-neurogenesis in the OB [18]. Thus these data indicate that, in order to more fully understand the role of adult-born neurons in odor memory tasks, there is a need for more detailed analyses that take into account the different steps of memory formation, execution, and storage.

One approach to address this issue would be to directly modulate the activity of adult-born neurons that are already integrated into the bulbar network. To achieve this, it is possible to take advantage of optogenetic activation or silencing of neurons, a method that has been used extensively over the last decade in many different fields of research [67, 68]. This technique enables the selective activation or inhibition of the activity of particular cell populations via expression at their membrane opsin receptors which are sensitive to light stimulation at a particular wavelength. Alonso and colleagues used this technique to investigate the involvement of adult-born interneurons in olfactory behaviour in the context of associative learning [17]. They showed that specifically activating adult-born neurons during learning of an operant olfactory discrimination task not only decreased the time necessary for the animals to learn the task, but also improved their memory of it. Animals in which adult-born neurons were activated were able to remember the task longer, as compared to control mice [17]. Other tools available to modulate the activity of different cellular populations are Designed Receptors Exclusively Activated by Designer Drugs (DREADDs), G protein-coupled muscarinic receptors genetically modified to respond exclusively to a pharmacologically inert molecule, clozapine-N-oxide (CNO) [69, 70]. When coupled to Gi family proteins, CNO treatment has an inhibitory effect on DREADD-expressing cells, whereas those coupled to Gq proteins are activated by CNO.

The tools presented above will enable investigation of the involvement of adult-born interneurons in olfactory behaviour during particular phases of odor task performance and there is urgent need for the implementation of these approaches to dissect the function of new cells in the OB.

## 4. Adult-Born Neurons: Distinct Functions for Different Subpopulation of Cells? 

Adult-born interneurons are produced in a region-specific manner and may be subdivided into different subsets based on the expression of neurochemical markers. Hack and colleagues showed that different subpopulations of adult-born neurons are produced in the region-specific manner in the SVZ and RMS [71]. Using retroviral labelling in these regions, the authors revealed that TH+ PGCs are generated in large numbers in the RMS [71]. Merkle and colleagues, by targeting stem cells, have further shown that both neonatally and adult-born cells in the OB are produced in a region-specific manner along SVZ-RMS pathway [72]. These data suggest that diverse subpopulations of interneurons are derived from a heterogeneous pool of stem cells.

The different subpopulations of adult-born neurons are produced not only in specific subregions, but also with a distinct temporal pattern throughout an animal lifetime. As briefly presented in the introduction, the GL contains at least three subpopulations of PGCs based on the expression of TH, calretinin, and calbindin [16, 73, 74]. Using dye labelling and homochronic/heterochronic transplantation, it has been shown that calbindin-positive PGCs are largely generated during neonatal life, whereas calretinin- and TH-expressing neurons are mainly produced during adulthood [75]. By contrast, using inducible genetic fate mapping of Dlx1/2 precursors, it has been shown that the production of TH-expressing PGCs is maximal during early embryogenesis [12]. More consensus exists for the temporal pattern of calretinin and calbindin-expressing PGCs production, as genetic fate mapping has revealed that the production of calbindin interneurons is maximal during late embryogenesis and declines postnatally, whereas calretinin cell production is low during embryogenesis and increases postnatally [12]. The different subpopulations of adult-born PGCs are responsive to odor stimulation as shown by* in vivo* two-photon Ca^2+^ imaging and targeted patch-clamp recordings [44, 48]. Moreover, their responses to olfactory stimulation are shaped in an experience-dependent manner [48]. However, the role of different subpopulations of adult-born PGCs in OB network function and odor behaviour remains unknown. Some morphofunctional differences are observed between different subtypes of PGCs indicating that they may play distinct roles in the OB. For example, it has been shown that TH-expressing PGCs are the only population of interneurons that receive input from the olfactory nerve [76]. By contrast, other populations of PGCs send their dendrites to intraglomerular zones that are not in contact with the sensory neuron's terminals [76]. Therefore, TH-expressing PGCs may be more sensitive to the level of sensory input. This is supported by observations showing that social odor perception after mating is associated with an increased level of TH expression in PGCs and dopaminergic transmission in the OB [77]. This increase in dopaminergic transmission upholds presynaptic inhibition of sensory neurons and hampers neuronal activation in the OB, thus leading to the modulation of social odor perception detrimental to pregnancy [77]. The relative contributions of adult-born and preexisting TH-expressing cells to this effect have yet to be studied, as have the roles of calbindin- and calretinin-positive PGCs in odor behaviour.

The OB also contains parvalbumin-expressing interneurons located in the external plexiform layer (EPL) [12, 78]. These interneurons are produced only during the perinatal period [12] and play an important role in feedback control of the OB output [78].

GCs are the largest population of neurons in the OB. Despite this, very little is known about neurochemical heterogeneity of these cells and how different subtypes of GCs shape odor information processing in the OB and olfactory behaviour. Indeed, until now essentially all of the studies investigating the role of adult-born neurons in olfactory behaviour have considered these cells as homogenous population of neurons [17–21]. It is conceivable, however, that each subtype of adult-born GCs plays a specific role in odor information processing. Among the different subtypes that have been described so far are calretinin-expressing GCs found in the superficial GCL; glycoprotein 5T4-expressing cells located in the mitral cell layer (MCL); and mGluR2-, CaMKIV-, and CaMKII-expressing ones found throughout the GCL [15, 79–81]. Of these, calretinin- and mGluR2-expressing neurons and at lower level 5T4-expressing cells have been shown to be renewed in adulthood [12, 14, 15], while the renewal of other subtypes have not been yet reported. Moreover, GCs with different morphologies to their early-born counterparts have recently been described as type 1 deep branching GCs, type 2 shrub GCs, type 3 perimitral GCs, and type 4 satellite cells [14]. What is the role of these neurons as well as other, as yet undiscovered, subtypes of GCs in odor information processing and odor behaviour? Interestingly, as with PGCs, some morphofunctional differences have been observed in GCs. While these data are still rudimentary, they imply that each subtype of GCs may play a distinct role in the OB. For example, it is well known that calretinin-expressing cells are located at the superficial GCL and may thus specifically contact tufted cells [12, 16]. Since tufted cells are involved in the synchronization of isofunctional odor columns in the OB [82] and GCs play a crucial role in the synchronization of principal cell activity [6–9], it is conceivable that calretinin-expressing GCs may be specifically involved in odor discrimination paradigms, especially those involving complex tasks based on the discrimination of similar odors. With regard to newly described subpopulations of adult-born GCs [14], some morphological differences were also documented. It has been shown that the dendrites of type 1 GCs do not reach the MCL or EPL but branch primarily in deeper regions of the OB. Type 2 shrub GCs extend a single primary dendrite into the deep layer of the EPL where they branch extensively, giving rise to many small dendrites decorated with numerous spine-like protrusions. The cell bodies of type 3 perimitral GCs are confined to the MCL, from which they extend thin spineless processes that branch above and below the MCL. Type 4 cells are located in the EPL and have branched dendrites with varicosities and few spines [14]. While the functional role of these new subtypes of GCs is not yet known, based on their morphology and location in the OB, it has been hypothesized that types 1 and 2 GCs may inhibit the cell bodies and proximal dendrites of principal neurons and thus mediate columnar inhibition and localized lateral inhibition, whereas types 3 and 4 cells may inhibit the output of principal neurons and their dendrites [14]. The above data demonstrate growing evidence for the heterogeneity of adult-born interneurons in the OB. While several subpopulations of PGCs and GCs have already been described, it is conceivable that other subpopulations will emerge in the near future. Some of this data implies that various subpopulations may differ functionally, something that must be studied further to increase our understanding of how olfactory information is processed in the OB. Hence, further investigation is needed to determine whether and how each adult-born (and preexisting) cell subpopulation makes a unique contribution to bulbar circuitry, and if so, whether there are preferential conditions under which their involvement is required. Moreover, if such differences can be revealed, it will be crucial to understand the impact of each subpopulation of interneurons on different olfactory behaviours.

## 5. Conclusion

Adult neurogenesis is an extraordinary process which constantly supplies the OB with new interneurons, a cell population that plays an important role in regulating information sent by principal cells to higher brain regions. This mode of maintaining the plasticity of the OB allows fine adaptation of the bulbar circuitry to the constantly changing environment. The question of the functional role played by these cells has to be better understood. In fact, studies aimed at determining the involvement of adult neurogenesis in different olfactory behaviours have produced dissenting data. There is therefore a need for a different approach to resolve this problem and the advent of new techniques over recent years will certainly aid in advancing our knowledge in this field of research. Moreover, the fact that different subpopulations of adult-born cells in the OB have been clearly identified implies that we can no longer consider neurogenesis as the birth of a homogenous population of interneurons but of different subsets that each makes a unique contribution to olfactory processing and network activity.

## References

[1] Shepherd G. M., Chen W. R., Greer C. A., Shepherd G. M. (2004). Olfactory bulb. *The Synaptic Organization of the Brain*.

[2] Isaacson J. S., Strowbridge B. W. (1998). Olfactory reciprocal synapses: dendritic signaling in the CNS. *Neuron*.

[3] Jahr C. E., Nicoll R. A. (1982). An intracellular analysis of dendrodendritic inhibition in the turtle *in vitro* olfactory bulb. *Journal of Physiology*.

[4] Price J. L., Powell T. P. (1970). The synaptology of the granule cells of the olfactory bulb. *Journal of Cell Science*.

[5] Whitman M. C., Greer C. A. (2007). Synaptic integration of adult-generated olfactory bulb granule cells: basal axodendritic centrifugal input precedes apical dendrodendritic local circuits. *Journal of Neuroscience*.

[6] Arevian A. C., Kapoor V., Urban N. N. (2008). Activity-dependent gating of lateral inhibition in the mouse olfactory bulb. *Nature Neuroscience*.

[7] Fukunaga I., Herb J. T., Kollo M., Boyden E. S., Schaefer A. T. (2014). Independent control of gamma and theta activity by distinct interneuron networks in the olfactory bulb. *Nature Neuroscience*.

[8] Urban N. N. (2002). Lateral inhibition in the olfactory bulb and in olfaction. *Physiology and Behavior*.

[9] Yokoi M., Mori K., Nakanishi S. (1995). Refinement of odor molecule tuning by dendrodendritic synaptic inhibition in the olfactory bulb. *Proceedings of the National Academy of Sciences of the United States of America*.

[10] Lagace D. C., Whitman M. C., Noonan M. A. (2007). Dynamic contribution of nestin-expressing stem cells to adult neurogenesis. *Journal of Neuroscience*.

[11] Ninkovic J., Mori T., Götz M. (2007). Distinct modes of neuron addition in adult mouse neurogenesis. *The Journal of Neuroscience*.

[12] Batista-Brito R., Close J., Machold R., Fishell G. (2008). The distinct temporal origins of olfactory bulb interneuron subtypes. *The Journal of Neuroscience*.

[13] Kosaka K., Toida K., Aika Y., Kosaka T. (1998). How simple is the organization of the olfactory glomerulus?: the heterogeneity of so-called periglomerular cells. *Neuroscience Research*.

[14] Merkle F. T., Fuentealba L. C., Sanders T. A., Magno L., Kessaris N., Alvarez-Buylla A. (2014). Adult neural stem cells in distinct microdomains generate previously unknown interneuron types. *Nature Neuroscience*.

[15] Murata K., Imai M., Nakanishi S. (2011). Compensation of depleted neuronal subsets by new neurons in a local area of the adult olfactory bulb. *Journal of Neuroscience*.

[16] Parrish-Aungst S., Shipley M. T., Erdelyi F., Szabo G., Puche A. C. (2007). Quantitative analysis of neuronal diversity in the mouse olfactory bulb. *Journal of Comparative Neurology*.

[17] Alonso M., Lepousez G., Wagner S. (2012). Activation of adult-born neurons facilitates learning and memory. *Nature Neuroscience*.

[18] Arruda-Carvalho M., Akers K. G., Guskjolen A., Sakaguchi M., Josselyn S. A., Frankland P. W. (2014). Posttraining ablation of adult-generated olfactory granule cells degrades odor–reward memories. *Journal of Neuroscience*.

[19] Breton-Provencher V., Lemasson M., Peralta M. R., Saghatelyan A. (2009). Interneurons produced in adulthood are required for the normal functioning of the olfactory bulb network and for the execution of selected olfactory behaviors. *Journal of Neuroscience*.

[20] Imayoshi I., Sakamoto M., Ohtsuka T. (2008). Roles of continuous neurogenesis in the structural and functional integrity of the adult forebrain. *Nature Neuroscience*.

[21] Sultan S., Mandairon N., Kermen F., Garcia S., Sacquet J., Didier A. (2010). Learning-dependent neurogenesis in the olfactory bulb determines long-term olfactory memory. *The FASEB Journal*.

[22] Alvarez-Buylla A., García-Verdugo J. M. (2002). Neurogenesis in adult subventricular zone. *The Journal of Neuroscience*.

[23] Doetsch F., Caillé I., Lim D. A., García-Verdugo J. M., Alvarez-Buylla A. (1999). Subventricular zone astrocytes are neural stem cells in the adult mammalian brain. *Cell*.

[24] Luskin M. B. (1993). Restricted proliferation and migration of postnatally generated neurons derived from the forebrain subventricular zone. *Neuron*.

[25] Lois C., García-Verdugo J.-M., Alvarez-Buylla A. (1996). Chain migration of neuronal precursors. *Science*.

[26] Snapyan M., Lemasson M., Brill M. S. (2009). Vasculature guides migrating neuronal precursors in the adult mammalian forebrain via brain-derived neurotrophic factor signaling. *The Journal of Neuroscience*.

[27] Whitman M. C., Fan W., Rela L., Rodriguez-Gil D. J., Greer C. A. (2009). Blood vessels form a migratory scaffold in the rostral migratory stream. *The Journal of Comparative Neurology*.

[28] David L. S., Schachner M., Saghatelyan A. (2013). The extracellular matrix glycoprotein tenascin-R affects adult but not developmental neurogenesis in the olfactory bulb. *Journal of Neuroscience*.

[29] Hack I., Bancila M., Loulier K., Carroll P., Cremer H. (2002). Reelin is a detachment signal in tangential chain-migration during postnatal neurogenesis. *Nature Neuroscience*.

[30] Saghatelyan A., De Chevigny A., Schachner M., Lledo P.-M. (2004). Tenascin-R mediates activity-dependent recruitment of neuroblasts in the adult mouse forebrain. *Nature Neuroscience*.

[31] Belluzzi O., Benedusi M., Ackman J., LoTurco J. J. (2003). Electrophysiological differentiation of new neurons in the olfactory bulb. *The Journal of Neuroscience*.

[32] Carleton A., Petreanu L. T., Lansford R., Alvarez-Buylla A., Lledo P.-M. (2003). Becoming a new neuron in the adult olfactory bulb. *Nature Neuroscience*.

[33] Panzanelli P., Bardy C., Nissant A. (2009). Early synapse formation in developing interneurons of the adult olfactory bulb. *Journal of Neuroscience*.

[34] Petreanu L., Alvarez-Buylla A. (2002). Maturation and death of adult-born olfactory bulb granule neurons: role of olfaction. *The Journal of Neuroscience*.

[35] Winner B., Cooper-Kuhn C. M., Aigner R., Winkler J., Kuhn H. G. (2002). Long-term survival and cell death of newly generated neurons in the adult rat olfactory bulb. *European Journal of Neuroscience*.

[36] Alonso M., Viollet C., Gabellec M.-M., Meas-Yedid V., Olivo-Marin J.-C., Lledo P.-M. (2006). Olfactory discrimination learning increases the survival of adult-born neurons in the olfactory bulb. *Journal of Neuroscience*.

[37] Corotto F. S., Henegar J. R., Maruniak J. A. (1994). Odor deprivation leads to reduced neurogenesis and reduced neuronal survival in the olfactory bulb of the adult mouse. *Neuroscience*.

[38] Mandairon N., Sacquet J., Garcia S., Ravel N., Jourdan F., Didier A. (2006). Neurogenic correlates of an olfactory discrimination task in the adult olfactory bulb. *European Journal of Neuroscience*.

[39] Moreno M. M., Linster C., Escanilla O., Sacquet J., Didier A., Mandairon N. (2009). Olfactory perceptual learning requires adult neurogenesis. *Proceedings of the National Academy of Sciences of the United States of America*.

[40] Rochefort C., Lledo P.-M. (2005). Short-term survival of newborn neurons in the adult olfactory bulb after exposure to a complex odor environment. *European Journal of Neuroscience*.

[41] Rochefort C., Gheusi G., Vincent J.-D., Lledo P.-M. (2002). Enriched odor exposure increases the number of newborn neurons in the adult olfactory bulb and improves odor memory. *The Journal of Neuroscience*.

[42] Saghatelyan A., Roux P., Migliore M. (2005). Activity-dependent adjustments of the inhibitory network in the olfactory bulb following early postnatal deprivation. *Neuron*.

[43] Yamaguchi M., Mori K. (2005). Critical period for sensory experience-dependent survival of newly generated granule cells in the adult mouse olfactory bulb. *Proceedings of the National Academy of Sciences of the United States of America*.

[44] Kovalchuk Y., Homma R., Liang Y. (2015). In vivo odourant response properties of migrating adult-born neurons in the mouse olfactory bulb. *Nature Communications*.

[45] Mizrahi A. (2007). Dendritic development and plasticity of adult-born neurons in the mouse olfactory bulb. *Nature Neuroscience*.

[46] Livneh Y., Mizrahi A. (2012). Experience-dependent plasticity of mature adult-born neurons. *Nature Neuroscience*.

[47] Livneh Y., Mizrahi A. (2011). Long-term changes in the morphology and synaptic distributions of adult-born neurons. *Journal of Comparative Neurology*.

[48] Livneh Y., Adam Y., Mizrahi A. (2014). Odor processing by adult-born neurons. *Neuron*.

[49] Cummings D. M., Snyder J. S., Brewer M., Cameron H. A., Belluscio L. (2014). Adult neurogenesis is necessary to refine and maintain circuit specificity. *The Journal of Neuroscience*.

[50] Lepousez G., Lledo P.-M. (2013). Odor discrimination requires proper olfactory fast oscillations in awake mice. *Neuron*.

[51] Kelsch W., Lin C.-W., Lois C. (2008). Sequential development of synapses in dendritic domains during adult neurogenesis. *Proceedings of the National Academy of Sciences of the United States of America*.

[52] Valley M. T., Henderson L. G., Inverso S. A., Lledo P.-M. (2013). Adult neurogenesis produces neurons with unique GABAergic synapses in the olfactory bulb. *The Journal of Neuroscience*.

[53] Belnoue L., Grosjean N., Abrous D. N., Koehl M. (2011). A critical time window for the recruitment of bulbar newborn neurons by olfactory discrimination learning. *The Journal of Neuroscience*.

[54] Magavi S. S. P., Mitchell B. D., Szentirmai O., Carter B. S., Macklis J. D. (2005). Adult-born and preexisting olfactory granule neurons undergo distinct experience-dependent modifications of their olfactory responses in vivo. *Journal of Neuroscience*.

[55] Nissant A., Bardy C., Katagiri H., Murray K., Lledo P.-M. (2009). Adult neurogenesis promotes synaptic plasticity in the olfactory bulb. *Nature Neuroscience*.

[56] Baker H., Morel K., Stone D. M., Maruniak J. A. (1993). Adult naris closure profoundly reduces tyrosine hydroxylase expression in mouse olfactory bulb. *Brain Research*.

[57] Bastien-Dionne P.-O., David L. S., Parent A., Saghatelyan A. (2010). Role of sensory activity on chemospecific populations of interneurons in the adult olfactory bulb. *Journal of Comparative Neurology*.

[58] Kelsch W., Lin C.-W., Mosley C. P., Lois C. (2009). A critical period for activity-dependent synaptic development during olfactory bulb adult neurogenesis. *The Journal of Neuroscience*.

[59] Lin C.-W., Sim S., Ainsworth A., Okada M., Kelsch W., Lois C. (2010). Genetically increased cell-intrinsic excitability enhances neuronal integration into adult brain circuits. *Neuron*.

[60] Breton-Provencher V., Saghatelyan A. (2012). Newborn neurons in the adult olfactory bulb: unique properties for specific odor behavior. *Behavioural Brain Research*.

[61] Corona R., Lévy F. (2015). Chemical olfactory signals and parenthood in mammals. *Hormones and Behavior*.

[62] Feierstein C. E. (2012). Linking adult olfactory neurogenesis to social behavior. *Frontiers in Neuroscience*.

[63] Lazarini F., Mouthon M.-A., Gheusi G. (2009). Cellular and behavioral effects of cranial irradiation of the subventricular zone in adult mice. *PLoS ONE*.

[64] Mouret A., Gheusi G., Gabellec M.-M., De Chaumont F., Olivo-Marin J.-C., Lledo P.-M. (2008). Learning and survival of newly generated neurons: when time matters. *The Journal of Neuroscience*.

[65] Lazarini F., Lledo P.-M. (2011). Is adult neurogenesis essential for olfaction?. *Trends in Neurosciences*.

[66] Mandairon N., Sultan S., Nouvian M., Sacquet J., Didier A. (2011). Involvement of newborn neurons in olfactory associative learning? The operant or non-operant component of the task makes all the difference. *Journal of Neuroscience*.

[67] Grosenick L., Marshel J., Deisseroth K. (2015). Closed-loop and activity-guided optogenetic control. *Neuron*.

[68] Zhang F., Wang L.-P., Brauner M. (2007). Multimodal fast optical interrogation of neural circuitry. *Nature*.

[69] Alexander G. M., Rogan S. C., Abbas A. I. (2009). Remote control of neuronal activity in transgenic mice expressing evolved G protein-coupled receptors.. *Neuron*.

[70] Armbruster B. N., Li X., Pausch M. H., Herlitze S., Roth B. L. (2007). Evolving the lock to fit the key to create a family of G protein-coupled receptors potently activated by an inert ligand. *Proceedings of the National Academy of Sciences of the United States of America*.

[71] Hack M. A., Saghatelyan A., de Chevigny A. (2005). Neuronal fate determinants of adult olfactory bulb neurogenesis. *Nature Neuroscience*.

[72] Merkle F. T., Mirzadeh Z., Alvarez-Buylla A. (2007). Mosaic organization of neural stem cells in the adult brain. *Science*.

[73] Kosaka K., Aika Y., Toida K. (1995). Chemically defined neuron groups and their subpopulations in the glomerular layer of the rat main olfactory bulb. *Neuroscience Research*.

[74] Kosaka T., Kosaka K. (2005). Structural organization of the glomerulus in the main olfactory bulb. *Chemical Senses*.

[75] De Marchis S., Bovetti S., Carletti B. (2007). Generation of distinct types of periglomerular olfactory bulb interneurons during development and in adult mice: implication for intrinsic properties of the subventricular zone progenitor population. *The Journal of Neuroscience*.

[76] Kosaka K., Kosaka T. (2007). Chemical properties of type 1 and type 2 periglomerular cells in the mouse olfactory bulb are different from those in the rat olfactory bulb. *Brain Research*.

[77] Serguera C., Triaca V., Kelly-Barrett J., Banchaabouchi M. A., Minichiello L. (2008). Increased dopamine after mating impairs olfaction and prevents odor interference with pregnancy. *Nature Neuroscience*.

[78] Miyamichi K., Shlomai-Fuchs Y., Shu M., Weissbourd B. C., Luo L., Mizrahi A. (2013). Dissecting local circuits: parvalbumin interneurons underlie broad feedback control of olfactory bulb output. *Neuron*.

[79] Imamura F., Nagao H., Naritsuka H., Murata Y., Taniguchi H., Mori K. (2006). A leucine-rich repeat membrane protein, 5T4, is expressed by a subtype of granule cells with dendritic arbors in specific strata of the mouse olfactory bulb. *Journal of Comparative Neurology*.

[80] Jacobowitz D. M., Winsky L. (1991). Immunocytochemical localization of calretinin in the forebrain of the rat. *Journal of Comparative Neurology*.

[81] Zou D.-J., Greer C. A., Firestein S. (2002). Expression pattern of *α*CaMKII in the mouse main olfactory bulb. *Journal of Comparative Neurology*.

[82] Zhou Z., Belluscio L. (2008). Intrabulbar projecting external tufted cells mediate a timing-based mechanism that dynamically gates olfactory bulb output. *The Journal of Neuroscience*.

